# On the Premonitory Symptoms of Cerebral Disease

**Published:** 1851-04-01

**Authors:** 


### ON THE PREMONITORY SYMPTOMS OF CEREBRAL DISEASE.

THE following important and interesting remarks, on some of the more prominent and characteristic symptoms of cerebral disease, are from the experienced pen of Dr. DEVAY, of Lyons, and published in the *Gazette Médicale de Paris* for January, 1851. This instructive memoir has been translated for our able and respected contemporary, the *London Journal of Medicine*, from whose pages we extract it.

"Those severe cerebral affections which rapidly terminate existence, and still more those which, before the fatal issue, gradually destroy the intellect, sensation, and motion, have been the object of much research. Notwithstanding numerous physiological experiments, vivisections, autopsies, minute examinations of the different degrees of consistence and colour of cerebral substance, &c., the knowledge of the physician has been but little enriched. The cranial vault offers an inflexible resistance to exploration; and this should impress on us the necessity of depending less on anatomy in the study of cerebral diseases. Instead of studying the brain when it is dead or dying, we should fix our attention on the unusual manifestations, and carefully collect the various aberrations, either of sensibility or of motion. If we cannot examine the brain by palpation, like other viscera, if we cannot auscultate it, as we do the heart or lungs, and thus seize on, by means of one or more of our senses, its successive degradations, we can, at least, detect is commencing affections, by observing the derangements of the functions over which it presides—the intellect, sensation, and motion.

"Researches of this kind embrace the premonitory symptoms of diseases varying in their anatomical relations; they take a view of all those affections which arise from an alteration in the brain, properly so called, from apoplexy to mental alienation. But even when, in a given case of cerebral disease, the elements are most favourable, a diagnosis can only be formed with reserve. Thus, when we expect to find softening, we may meet with induration of the cerebral substance; we may expect to find tubercles, and discover hydatids, or osseous tumours. The phenomena presented by patients have not then always that conformity which would permit particular symptoms to be accurately referred to certain determinate forms of pathological affections. This clinical difficulty in the appreciation of the symptoms of confirmed disease, exists in an equal degree with regard to the interpretation of the premonitory symptoms. These may point to a functional derangement of the encephalon, without any anatomical lesion; and, when the latter is present, it may vary in its form. The infinite variety of forms, presented by the symptoms of a cerebral disease, can no more be explained by the various conditions in which the organ is found, which has been their seat, than the different modes, in which the same function is performed in different individuals, can be accounted for by anatomical differences in the part which is its instrument. Nevertheless, the study of premonitory symptoms may be useful, even for the anatomical diagnosis of the affection.

"After having devoted a considerable number of years to the attentive observation of the severe affections of the brain, we have become convinced, that there *most frequently* exists, especially in those which have a chronic course, a precursory stage, the signs in which are the diminutive of those symptoms which will, at a later period, constitute the more advanced degree of the disease. Thus, slight want of power in the lower limbs, and defect of precision in certain movements, represent paraplegia, or complete paralysis; and a slight alteration of the intellect bears the same relation to the delirium following it. In certain cases, without doubt, a sudden effusion of blood, breaking down the cerebral structure, may suddenly cut off an individual who a few moments before seemed in perfect health; in this case we admit that there has been no intermediate stage—that the index to the explosion has not existed. But this is by no means the case in a very great number of other forms of apoplexy, in which, as may be easily proved by examples, the precursory symptoms, denoting cerebral lesion, may continue for a long time. These forms of cerebral hæmorrhage enter then into the category of those severe affections of the brain—softening, induration, mental alienation, &c.—where a hidden molecular change has been going on before they have declared themselves. Andral, in speaking of certain premonitory symptoms of cerebral hæmorrhage, says: 'Their existence incontestably proves that, before the blood is effused, there is already a morbid action going on in the brain, the nature of which it would be important to determine.'

"I. Premonitory Signs, furnished by the Intellectual and Moral Faculties.—Almost all authors of repute have mentioned, without always attaching much importance to them, the disturbances of intellect which precede attacks of severe cerebral ideas. M. Gendrin says: 'Apoplectic attacks are often preceded by a difficulty in undertaking intellectual work, by an incapacity for unusual attention, by an extraordinary irascibility, by a morose weakness which exaggerates impressions, and produces terrors without a cause, or by unreasonable anxiety concerning ourselves, or those related to us.'* Insanity also has its period of incubation, its premonitory symptoms; and frequently it is found that the first act of insanity which caused alarm, has been preceded by several symptoms which had escaped observation, and sometimes the first phenomenon of the disease has been taken for its cause. The insane often combat their false ideas, before the disorder of their reason, and the internal contest which precedes the explosion of their madness, are perceived.† The most general precursor of every severe affection of the brain is a state of *cerebral lassitude*, presenting much analogy to that state of intellectual torpor which follows severe or pestilential fevers. There is observed in the habitual gesture of the patients, in their attitudes and movements, a total absence of what may be called the consciousness of action. The brain seems to have lost its *balancing* power over the *ensemble* of the functions of the life of relation. These patients are often in a constant state of slight habitual vertigo, which they call *weakness of the head*, and which is frequently accompanied by debility in the limbs.

"The *memory* is frequently impaired in the precursory period of cerebral affections. Thus, patients have forgotten the names of their friends, or of the most common things. In conversation, they have difficulty in finding the proper word to express their meaning, and are obliged to make use of circumlocutions. More rarely, the memory becomes more powerful; it seems to take a new flight, and reproduces, to the great astonishment of the patient and his attendants, events which had seemed to be entirely forgotten. The curious and inexplicable fact *of reminiscence* corresponds to the exaltation of the special sensibility of certain senses. It is sometimes observed after a slight attack of apoplexy. Professor Brachet has communicated the case of a man, aged 50, who was attacked with apoplexy: he preserved his speech, but could only express himself correctly in the *patois* of his country, which he had entirely forgotten while in health.

"Next to the impairment of the memory, and also of the attention, which is fixed with difficulty, or not at all, on objects presented to the notice of the individual, the most striking change is in *volition*, which is diminished. The man who has hitherto been most firm, who has shown most tenacity in his views, who has pursued the plan of his life with great determination, becomes, in a measure, like the toy of a child; those who are about him, even his inferiors, can command him. Human depravity has often taken advantage of this moral decadence for culpable ends; and the man who has hitherto most rigorously and carefully managed his affairs, is all at once spoiled of his goods, either by extorted donations, or by burdensome expenses. The public see in these cases *bizarreries* of character; the physiologist and the physician see in them the first expression of a pathological condition. This weakening of the will, which, according to our observations, is chiefly connected with those cerebral lesions which lead to lunacy, or to paralysis of the insane, necessitates an alteration of the judgment. . . . . The will is the result of the other faculties; and it is not because it is wanting in the idiot, or lunatic, that they are irresponsible; but rather because they are ignorant of the rules which should direct it.

"There is but a slight transition from this to *perversion of the moral faculties*—one of the most mysterious points in psychology. It would seem as if the balancing power of the brain, which regulates the locomotive movements, were also defective as regarded the moral part of the man, so that he fell under the empire of instinct. Hence arises degradation of ideas, and lascivious conversation, in persons who have hitherto appeared full of decency and modesty. And this vitiation of the moral faculty may break through the sphere of theory, and become realized in action. This fact is important in a medico-legal point of view. . . . .

---

* Gendrin, "Traité philosophique de Médecine pratique," tome i. p. 487.

† Esquirol, "Des Maladies Mentales," t. i. pp. 77, et suivant. Dr. Forbes Winslow on the "Incubation of Insanity," in Trans. of Med. Soc., Lond., vol. i. N. Series.

"The already important and difficult question of moral responsibility will become more delicate, under the supposition of a commencing affection of the organ of thought. When a person of hitherto irreproachable character commits a reprehensible action, the physician is disposed to plead extenuating circumstances, not because the case demands it, but for the sake of human nature, knowing all the aberrations of which it is susceptible. Experience also should be invoked, as well as reason, in deciding on so delicate a case.

"I am now attending a woman, aged 42, who for a year and a half has gradually fallen into a state denoting general softening of the brain: almost entire blindness, inability to walk, semi-stupid intellect, &c. &c. Two years ago, she only felt severe and almost constant pain in the head; her general health was in other respects perfectly good, her intellect clear. Three years ago, this woman, though possessed of a competency, *committed a petty theft in a fair.* Dr. Brierre de Boismont, in his observations on 'General Paralysis of the Insane,' states that this disease is preceded by a premonitory period, for about six or seven years, or more, before the apparent explosion of the insanity. There are *perversions of the moral and effective faculties*, without less ability on the part of the individuals presenting these changes to fulfil the duties of social life, or to perform their functions. The acts of indecency, of dishonesty, of debauchery, like which there had been nothing before, are suppressed and compensated for; then at last the patient is seized with symptoms of general paralysis.

"'A person high in office,' says Dr. Brierre de Boismont, 'had performed the duties of his station up to the time when I was consulted; and yet the details, which were furnished to me by his wife, left no doubt that his moral and affective faculties had been for some time impaired. From having been generous and honest, he had, for more than six years, exhibited a degree of sordid avarice and unbridled licentiousness. With the progress of the disease, his avarice was manifested in mean actions; he refused to pay his debts, maintaining that he had already done so; and even purloined objects from the houses of his acquaintances. Until the last-named acts were committed, no one had suspected that his mind was disordered..... Some time after, I was called in consultation to see a retired public officer, whose thefts had made much noise some years previously. The particulars with which I was furnished regarding this interesting subject, made me then think that this person was labouring under the premonitory symptoms of general paralysis; I was almost sure that I should see a paralytic lunatic. The first words which he uttered in my presence showed me that the affection was far advanced. His delinquencies had been noticed eight years before; and it was only a few months ago that mental alienation was recognised.'*

"Dr. Passot has recently observed a case, in which delirium tremens appears to have been the proximate cause of the moral disturbance which supervened at a later period. Although differing in some circumstances, and although the proof *à posteriori*, that is, by the termination, has not yet been furnished, this case appears to have some resemblance to those related above. A cooper, aged 34, previously of irreproachable character, and enjoying a high reputation for honesty, was seized with delirium tremens, from which he recovered. But from this moment his conduct was deranged; he borrowed money from all quarters, and denied having ever received it. At last, after having cheated many, he fled to avoid prosecution. His intellect appeared sound; but Dr. Passot remarked, that he gave proofs of a considerable impairment of judgment, by asking persons for money from whom he had already borrowed.

"These and similar examples show what difficulties are presented in this new point of view of the doctrine of moral responsibility, and how much the question requires to be elucidated by an attentive study of the precursory symptoms of encephalic affections. It may be—and there is no physiological improbability in the supposition—that an immoral or obscene action is as abrupt and unexpected an occurrence as an aberration of the senses; one of those irregular paralytic affections which almost infallibly denote an approaching disorganization of the nervous centres. If there is a concomitance between the two occurrences, the aggregate of the pathological symptoms may be considered as furnishing evidence in favour of the non-culpability of the subject. But unfortunately the proof is often furnished only when the disease is confirmed—when the cerebral symptoms are already strongly marked. It will then be easy for the physician to trace the connexion between the previous act of the patient, and the symp-

* "Gazette Médicale de Paris," 1847, p. 393.

toms which now appear; but will not the patient have already suffered the rigorous application of penal laws? This is a delicate problem, for the solution of which a most careful exercise of the conscience and knowledge of the physician is required. If any one is called on to give his opinion to enlighten justice regarding an infraction of morality, *committed without precedents*, in a moment when the individual is in full possession of the faculties of relation, he should express such opinion with the greatest reserve. It will be for him to institute a searching and severe inquiry into the previous condition of the patient, his attitudes, his sleep, his will, his memory, his sensation, &c.; and perhaps he may then be able to discover some sign, from which he may deduce the irresponsibility of the subject.

"The abrupt changes which may occur in a man's tastes, in his inclination, in his manner of living, in a word, in his social aspect, are worthy of attention. Modifications of this nature, when they do not appear in a slow and progressive manner, do not arise from the action of moral influences, and can only arise from a change in the nervous system. Thus it has long been remarked, that unusual gaiety in an habitually grave individual may denote the approach of an attack of apoplexy. It is the same with those who suddenly seek for noise and bustle, after having loved retirement and quietness for a great part of their life. We have known a man, aged 57, who, having up to that time led a grave and even austere life, gave himself up to the pursuit of amusements unsuited to his age, and was, a few months after, seized with sudden and complete apoplexy (*apoplexie foudroyante*). In this case, which we observed a few years ago, we were led to form an unfavourable prognosis. A man most estimable for mental endowments, and for the qualities of his heart, came one day to converse with us on subjects not relating to his health. His conversation was clear; nothing was indicated in his gait; but he had for some time complained of inaptitude for work. While we were occupied in writing a letter, we saw him rise, rummage a drawer, and open a note. This act, on the part of a person of the most polite and discreet habits, struck us forcibly. We connected it with two other circumstances which were known to us. During the revolution of February, this gentleman, holding an important post in the administration, had engaged, from the most disinterested and praiseworthy views, in public agitation, from which his mind had received a strong impression: his mother had also been attacked with senile dementia. Three months after, the patient lost his sight after violent headaches, and he subsequently died, with all the symptoms of cerebral softening. A complete change in the turn of the ideas, when it is not the result of advanced age, when it manifests itself in a short period of time, and when it cannot be traced to the action of moral influences, is very suspicious. We have known a young physician, who exhibited this phenomenon in a very marked manner, and who, a short time after, was seized with paralysis of the insane. When we knew him three years before, he was very free in his assertions, and inclined to exaggerate; but he had become discreet, and wary in his speech. His former condition, and the medium in which he had lived, showed sufficiently that this change could not be the effect of a *progressive amendment;* we considered that there was some disease, and our opinion was ultimately confirmed.

"It is conceivable that the same psychological perturbation which changes the moral sentiments may likewise impair the sentiment of self-preservation; and hence that *suicidal melancholy* may mark the commencement of a severe affection of the brain. This disease is, moreover, very often conjoined with a lesion of the intellectual and affective faculties.

"II. Premonitory Signs furnished by the Sensorial Functions.—Most of these are furnished by the sense of *vision*. We will merely mention dimness, the appearance of objects as if coloured red, photophobia, &c., which may indicate threatening meningitis, as well as cerebral hyperæmia; these symptoms bear an especial relation to acute diseases of the encephalon. These signs may exist several years before the explosion of the disease. Before attacks of apoplexy, impairment of vision sometimes exists in a high degree without being known to the patients, especially when, as is most commonly the case, it is not sufficient to prevent them from seeing those who are about them. The mistake is the more easy, as this symptom may be limited to one eye; the other compensating for the weakness of its fellow. Amblyopia is a frequent symptom; sometimes there is complete blindness, as in the case of the Baron Hornestein, cited by Wepfer (*Anatomia Apoplecticorum*), who became blind three weeks before a fatal attack of apoplexy.

"A valuable sign, belonging in some degree to what may be called the expression of the eyes, consists in a want of parallelism in these organs; it is not squinting, nor is it the look of hallucination. It seems pretty well defined by the following expression: *The eyes are not in the axis of the reason.* There may be certain defects in this relation pointed out between a material object and a moral fact; but those persons who are accustomed to scrutinize the human look, and to see reflected in it the different passions, will easily understand me.

"The phenomenon of exaltation of special sensibility, as a precursory sign of a severe encephalic lesion, is sometimes met with. It is in this case, as in other circumstances in which it is observed, one of the most mysterious problems for the physiologist.* It is well known that hearing often becomes excessively acute before attacks of apoplexy. The patients, incommoded by the least noise, become irascible; they perceive distant sounds, which are unheard by those who are with them. This fineness of hearing must be distinguished from the perception of strange and imaginary sounds, which is nothing but a sensorial hallucination.

"The following is a case in which disease of the brain was first indicated by enlargement of the field of vision.

"Case.—A painter, aged thirty-two, was admitted in 1849 into the Hôtel-Dieu at Lyons. This young man, who was possessed of some talent, had been gradually reduced to distress, partly by political disturbances, partly by other causes. A year before entering the hospital, his sight, which was previously good, acquired greater development; from his window, which opened into a very long street, he could distinguish objects and persons whom he could before neither distinguish nor even see. This circumstance troubled him, and surprised those about him. The exaltation of vision continued until August 1848, when he was seized with violent continued pains in the right parietal region; at this time there was slight weakness in the left arm. The symptoms increased till March 1849, when there was paralysis and contraction of the right arm, and blindness of the left eye. When he entered the hospital in July, the following was his condition. There was almost complete stupor; the paralyzed eye was almost completely covered by the upper eyelid; there was paralysis, with contraction, of all the left side of the body; the urine and fæces were discharged involuntarily. He continued in this state until the beginning of September, when death ensued, preceded by symptoms of slow fever. The autopsy revealed partial circumscribed softening of the middle and upper part of the right hemisphere, for the extent of about two *centimètres;* the convolutions were pale and puffy; the pulp was diffluent, and of a dirty grey colour. Except the corpus callosum, which appeared soft, the rest of the cerebral substance was sound.

"This phenomenon, judging from a passage in the writings of Andral, seems to have been observed in other cases. 'Cases have been observed in which, for a longer or shorter period before the attack, the sight has acquired an unusual degree of fineness. The existence of these important phenomena, which are often presented by vision at a longer or shorter period before the occurrence of hæmorrhage, prove incontestably that, before the blood is effused, there is already some morbid action, either continuous or intermittent, in the brain, of which it would be important to determine the precise nature.'†

"The sense of *hearing* may present the same modifications as that of vision. Some persons are tormented with drumming in the ear, with continued or intermittent tinkling. Some believe that they hear the most strange noises. These hallucinations are by no means the constant precursors of an encephalic attack; they may be connected with simple perversions of the sensorial function.

Premonitory Signs furnished by the Organs of Motion and Sensation.—The alterations in the *muscular functions* present great variety, from the simple hesitation which we have already noticed, to paralysis which is complete, but which, on account of its nature and its seat, we shall denominate *irregular paralysis.* It is not uncommon to observe a state of general languor which makes the patients seek for rest—for the *far niente.* Van Swieten has remarked, in treating of apoplexy: *Primò*

---

* See the *feuilleton* of the "Gazette Medicale" for 1848, tome iii. p. 41, where several cases of exaltation of the senses are related.

† Andral, "Clinique Médicale," tome v.

*oritur languor et amor quietis et otii.* At other times, those who are about to be attacked with cerebral disease are much agitated, and expend a great amount of activity in their movements. Dr. Tessier has lately attended a lady, aged 60, who from the critical age, has been subject to attacks every month, at the period when she used to menstruate. She loses consciousness; and, after having recovered her senses, is paralyzed on one side of the body, with great embarrassment of speech. These symptoms continue some days, and gradually leave her, to return at the fixed period. But some days before the new attack, this lady, though usually quiet and peaceable, exhibits much agitation; she cannot remain in her place, and those who are about her always know what this sign means. In this case, we recognise an example of *periodic nervous apoplexy.*

"Impairment of muscular motion is exhibited in various degrees. It is especially remarked in the lower limbs, which seem to bend under the weight of the body, and render the gait rather unsteady. This debility is the more striking if the person be young, and has no apparent cause for it. Portal was able to prognosticate an attack of apoplexy in a gentleman apparently in perfect health, from observing a slight fixedness in the left eye and a slight weakness in the leg of the same side. *The digitus semi-mortuus,* noticed by Dr. Marshall Hall, is one of those instances of *irregular paralysis,* of which it is so important to determine the true signification. Some time ago, we saw the following case. A man, aged 54, one day called on us. In conversation, he jokingly noticed a sort of deadness which he felt in the little finger of the left hand, while the rest of the hand was able to perform its ordinary functions. We advised him to put himself under treatment: he neglected this advice, and some days after was seized with cerebral congestion, which left his faculties remarkably weakened. The *digitus semi-mortuus* has shortly since been noticed in a valuable communication from Dr. Gillet de Grandmont.

"Irregular paralyses, which seem to arise from exhaustion of the sources of the sensitive and motor powers, may appear under circumstances in which they do not constitute a symptom of such great importance. Such are those which sometimes follow hysterical convulsions, lead-colic, venereal abuses, &c. Here, these phenomena are connected with *transient* modifications of innervation. The suddenness of the attacks, their frequent isolation from other symptoms, their seat in parts distant from each other, while those lying between preserve the integrity of their movements, constitute the exceptional characters of those palsies which are connected with a latent alteration in the nervous centres. We must not lose sight of the difficulty of deglutition which some patients experience some time before being attacked: as well as the semi-paralysis of the vocal cords and tongue, giving rise to stammering or aphonia. The paralysis of the upper eyelids, which become œdematous, is also a sign of great value.

"*General sensibility* may be abolished, simply diminished, or exaggerated. The two first forms almost always follow muscular paralysis; but they may exist alone. Sensibility may be exaggerated in two forms. The patients may present hyperæsthesia, or exquisite sensibility of the whole cutaneous surface; so that the least touch troubles them. This is an increased anormal sensibility—an exaggeration of the sense of touch, corresponding to the exaltation of the sensorial faculties which we have already studied. Sensibility may also be exalted in the form of pain; and this merits our most careful attention. Violent pains, precursory of a severe cerebral lesion, have often been mistaken for neuralgia. The same is the case in treating cephalalgia, supposed to be dependent on dyspepsia: and this error is more readily fallen into, as the stomach is often disordered. The diagnosis in these cases is sometimes difficult; but the duration and violence of the pain will lead to the suspicion, that there is something more than ordinary headache, and that, although the functions of the stomach are troubled at the same time, the headache is often too intense to be accounted for by the state of that organ. The patient cannot in general endure a warm room, nor the noise made by persons about him, nor even the fatigue of agreeable conversation, without suffering an aggravation of his headache. The paroxysms are sometimes accompanied with vomiting, and sometimes with violent beating in the head. If with these symptoms we remark paleness of face and weakness of pulse, and if active measures have been employed without benefit, we are led to suspect the presence of organic lesion.* Painful cramps are not unfrequent. Portal has seen patients who suffered severely from cramps in the legs before an attack of apoplexy.

* Abercrombie, "Diseases of the Brain," p. 453.

"Cutaneous sensibility presents other singular modes of perversion. A case is related of a man who, several months before being attacked with apoplexy, experienced from time to time an absolute loss of sensibility on five or six isolated points of the skin of the thorax, each of about the size of a five-franc piece. Here the skin might be pinched without causing any pain; beyond, the sensibility was perfect. These partial abolitions of sensation were not constant. On some days there was not the least diminution of sensibility; then suddenly, and simultaneously, it was annihilated in the isolated portions. Such unusual modifications of functions directly dependent on the brain, ought to furnish us with arguments in favour of the possibility of moral and instinctive perversions, and of their dependence, not on the corruption of the moral faculty itself, but on a latent pathological condition of the organ. Hence arises the doctrine of irresponsibility.

"It is in the life of relation that indicatory signs are especially to be looked for. At the initial period of severe cerebral disease, organic life reveals few or no disturbances. The symptoms which may exist under the head only acquire value in connexion with those which are derived from the life of relation. The brain must be much affected to produce changes in the nutritive function. Excepting sleep, which is on the confines of animal and organic life, there is not in the latter any essential functional disturbance. In the initial period, most patients have lost the power of sleep, or, if this function be performed, it is rather a fatiguing drowsiness than refreshing sleep. The digestive functions present no other special disorder than obstinate constipation, which is often difficult to be overcome by drastics. The eyelids sometimes become œdematous; and, in some subjects, attacks are preceded by small effusions of blood, even in the tissue of the conjunctiva. The secretions are but little altered. The urine is sometimes highly albuminous; but this is a subject for further researches.

"In subsequent communications, Dr. Devay proposes to treat of the etiology and treatment of incipient cerebral affections."

